# Prospects of perfusion contrast-enhanced ultrasound (CE-US) in diagnosing axillary lymph node metastases in breast cancer: a comparison with lymphatic CE-US

**DOI:** 10.1007/s10396-024-01444-w

**Published:** 2024-04-20

**Authors:** Naoko Mori, Li Li, Masazumi Matsuda, Yu Mori, Shunji Mugikura

**Affiliations:** 1 Department of Radiology, Akita University Graduate School of Medicine, 1-1-1 Hondo, Akita, Akita 010-8543 Japan; 2 Department of Diagnostic Radiology, Tohoku University Graduate School of Medicine, 1-1 Seiryo-Machi, Aoba-Ku, Sendai, 980-8574 Japan; 3 Department of Orthopaedic Surgery, Tohoku University Graduate School of Medicine, 1-1 Seiryo-Machi, Aoba-Ku, Sendai, 980-8575 Japan; 4 Division of Image Statistics, Tohoku Medical Megabank Organization, Tohoku University, Sendai, Japan

**Keywords:** Breast cancer, Lymph node metastasis, Ultrasound, Contrast-enhanced ultrasound

## Abstract

Accurate diagnosis of lymph node (LN) metastasis is vital for prognosis and treatment in patients with breast cancer. Imaging 1modalities such as ultrasound (US), MRI, CT, and 18F-FDG PET/CT are used for preoperative assessment. While conventional US is commonly recommended due to its resolution and sensitivity, it has limitations such as operator subjectivity and difficulty detecting small metastases. This review shows the microanatomy of axillary LNs to enhance accurate diagnosis and the characteristics of contrast-enhanced US (CE-US), which utilizes intravascular microbubble contrast agents, making it ideal for vascular imaging. A significant focus of this review is on distinguishing between two types of CE-US techniques for axillary LN evaluation: perfusion CE-US and lymphatic CE-US. Perfusion CE-US is used to assess LN metastasis via transvenous contrast agent administration, while lymphatic CE-US is used to identify sentinel LNs and diagnose LN metastasis through percutaneous contrast agent administration. This review also highlights the need for future research to clarify the distinction between studies involving “apparently enlarged LNs” and “clinical node-negative” cases in perfusion CE-US research. Such research standardization is essential to ensure accurate diagnostic performance in various clinical studies. Future studies should aim to standardize CE-US methods for improved LN metastasis diagnosis, not only in breast cancer but also across various malignancies.

## Introduction

Diagnosis of lymph node (LN) metastasis in patients with breast cancer is essential in predicting the prognosis and determining the treatment strategy. The presence and number of axillary LN metastases correlate significantly with overall survival and distant recurrence-free survival [1]. A higher number of LN metastases is associated with a higher recurrence rate [2]. For management, core needle biopsy or fine-needle aspiration is indicated if LN metastasis is suspected based on preoperative imaging evaluation [3]. The surgical strategy, whether to perform sentinel LN biopsy (SLNB) or axillary LN dissection, depends on the presence or absence of metastases on preoperative imaging evaluation. Neoadjuvant chemotherapy may be indicated when LN metastases are apparently observed on preoperative imaging evaluation [4].

The preoperative imaging evaluation of axillary LNs generally includes ultrasound (US), magnetic resonance imaging (MRI), computed tomography (CT), and 18F-fluorodeoxyglucose positron emission tomography–computed tomography (18F-FDG PET/CT) [4, 5]. In patients with breast cancer, increased LN short diameter and loss of fat at the hilum are findings suggestive of metastasis on modalities such as CT and MRI [6–8]. The increased LN short diameter and loss of fat at the hilum may be features that appear when metastatic sites are large. Early LN metastases are found in LNs without deformation of shape or size, and it may be difficult to detect early LN metastasis based on shape or size [9]. Conventional US (non-contrast) has higher resolution and better sensitivity than CT, MRI, or 18F-FDG PET/CT and is recommended as a primary modality for preoperative imaging evaluation of axillary LNs [3]; conventional US can reliably detect metastatic deposits larger than 5 mm and can also detect LN metastases smaller than 5 mm [4]. On the other hand, conventional US has the disadvantage of its operator-dependent subjective nature and inability to detect very small metastases that do not change the morphology of the LNs.

**Fig. 1:**
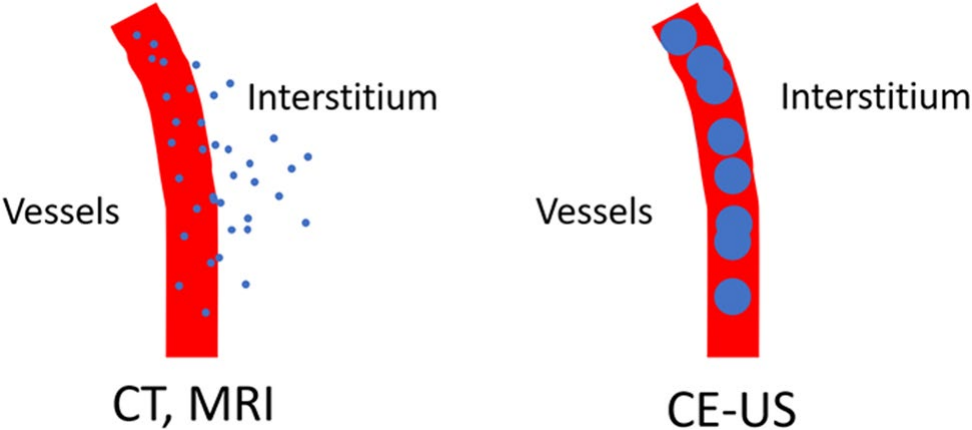
Schema of contrast agents for contrast-enhanced ultrasound (CE-US) compared to other modalities. The contrast agent used in computed tomography (CT) and magnetic resonance imaging (MRI) has a small particle size and is imaged when it leaks from blood vessels into the interstitium. In contrast, the contrast agent used in CE-US has a large particle size of 3–5 μm in diameter and remains in the blood vessels

Contrast-enhanced US (CE-US) has been clinically available for the assessment of focal liver lesions worldwide, and it is indicated for breast tumors in some countries, such as Japan [10, 11]. US contrast agents are composed of small volumes of air or gas surrounded by a stabilizing shell that remains completely intravascular, making them ideal agents for vessel imaging (Fig. 1) [12, 13]. CE-US is recommended for evaluating focal liver lesions that are inconclusive on CT or MRI and for detecting liver metastases as part of a multimodality imaging approach [14]. In the case of breast tumors, the European Federation of Societies for Ultrasound in Medicine and Biology (EFSUMB) states that a specific pattern indicative of malignancy has not been identified on CE-US. It cannot be recommended for use in routine clinical practice [15]. However, it is an important research topic as Miyamoto et al. reported the usefulness of CE-US using Sonazoid in distinguishing breast masses in 2014 [16].

While there are negative recommendations of CE-US for breast cancer, there are many studies on CE-US for the diagnosis of axillary LN metastasis of breast cancer. The purpose of CE-US in the diagnosis of axillary LN metastasis in patients with breast cancer can be divided into two categories: (1) identification of sentinel LNs and diagnosis of LN metastasis via percutaneous contrast agent administration (lymphatic CE-US) and (2) diagnosis of LN metastasis via transvenous contrast agent administration (perfusion CE-US). Since LNs have both lymphatic and blood perfusion, percutaneous or transvenous injection of contrast agents can rapidly reach the LNs by different paths [17, 18]. The purpose of this review is to shed light on the current status of perfusion CE-US for the diagnosis of axillary LN metastasis and to consider the future prospects of perfusion CE-US, leaving lymphatic CE-US for a separate chapter [19].

## Microanatomy of axillary LNs to understand the mechanism of detecting LN metastasis using CE-US

**Fig. 2:**
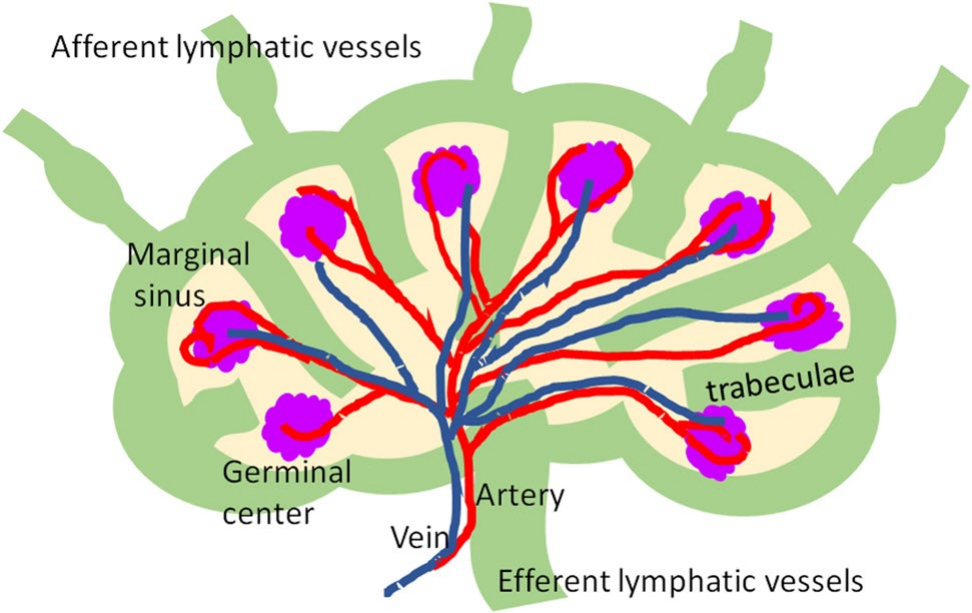
Schema of microanatomy of lymph nodes (LNs). Lymphatic fluid enters the marginal sinuses through the afferent lymphatic vessels on the capsular side and exits through the efferent lymph vessels on the lymph hilum side. The arteriovenous vessels are arranged in bundles from the lymphatic hilum to the cortex, and the cortex is nourished by branched capillaries from the arteriovenous vessels of the hilum

The surface of LNs is covered by a capsule, and the internal parenchyma is a network of cells with numerous T and B cells that provide immunity. Antibody production and collection of antigenic information by these T and B cells are performed within the LNs. Lymphatic fluid enters the marginal sinuses through the afferent lymphatic vessels on the capsular side and exits through the efferent lymph vessels on the lymph hilum side (Fig. 2) [20]. Therefore, it is generally accepted that early metastases occur initially in the subcapsular marginal sinus. Arteriovenous vessels that carry blood to and from the LNs are located around the efferent lymph vessels, and these are called the lymphatic hilum (Fig. 2). The construction of blood vessels within LNs has long been studied. In the 1980s, Morton et al. reported the presence of flow signals at the lymphatic hilum using color Doppler imaging [21]. The vascular architecture within the LNs was then examined using micro-angiogram techniques, which showed that arteriovenous vessels are arranged in bundles from the lymphatic hilum to the cortex, and that the cortex is nourished by branched capillaries from the arteriovenous vessels of the hilum [22, 23]. In the 2000s, several groups reported that histopathological microvessel density in metastatic LNs was low compared to non-metastatic LNs [24, 25]. On the other hand, animal studies have shown the appearance of a hypervascular response near the metastatic nests [26, 27]. The difference between the two results, i.e., low microvessel density vs. hypervascular response, may be due to the difference in the timing of the observed LN metastases, the latter of which may have appeared hypervascular due to advanced LN metastases.

## CE-US for assessment of LN metastasis

Tschammler et al. classified the pattern of the vascular architecture in normal and metastatic LNs using color Doppler imaging and showed that the abnormal vascular architecture within the LNs and increased blood flow surrounding the LNs might be findings suggestive of LN metastasis [36]. Non-hilar blood flow is speculated to reflect disruption of the normal blood flow from the lymphatic hilum due to metastasis within the LNs and the dilatation of surrounding vessels. These findings on color Doppler imaging, a non-contrast flow imaging technique, have led to the current assessment of the vascular architecture of LNs using CE-US, a contrast-enhanced flow imaging technique. Several studies have attempted to detect LN metastasis using perfusion CE-US, and in addition to axillary LN metastasis in breast cancer, perfusion CE-US has been used to evaluate LN metastasis in other areas, such as thyroid cancer. As described in Chapters 2 and 3, in vivo histopathological studies have shown that a decrease in microvascular density occurs at the site of metastasis, and animal studies have revealed an increase in microvascular density adjacent to metastasis within LNs. Perfusion CE-US may be useful for detecting changes in microvascular density due to metastases within LNs. We describe below the characteristics of contrast agents in perfusion CE-US, development of animal models of LN metastasis and perfusion CE-US imaging, differences between perfusion CE-US and lymphatic CE-US in the diagnosis of axillary LN metastases in breast cancer patients, diagnostic performance of perfusion CE-US for LN metastasis, and application of perfusion CE-US for diagnosis of LN metastasis in other areas.

### 2–1. Characteristics of contrast agents in CE-US

Microbubbles (product name: Sonazoid (Daiichi Sankyo Company, Limited)) are a second-generation ultrasound contrast agent consisting of gas (perflubutane) (diameter of 3–5 μm) coated with egg white [28] that has been covered by health insurance for breast cancer in Japan since 2012 [16, 29, 30]. Because of their size, the microbubbles used in perfusion CE-US cannot leak into the interstitium through vascular endothelial spaces, unlike CT or MRI contrast agents (Fig. 1), and thus enables ideal vessel imaging [12]. Taking these characteristics into account, a prospective, open-label, multicenter phase 3 study showed that perfusion CE-US with Sonazoid was superior to non-contrast US in the differentiation of local breast lesions (benign or malignant)[16]. Furthermore, perfusion CE-US is expected to allow evaluation of not only local breast lesions but also axillary LNs by allowing visualization of microvessel density, and research on perfusion CE-US in the diagnosis of axillary LN metastasis continues.

### 2–2. Development of animal models of LN metastasis and perfusion CE-US imaging

**Fig. 3:**
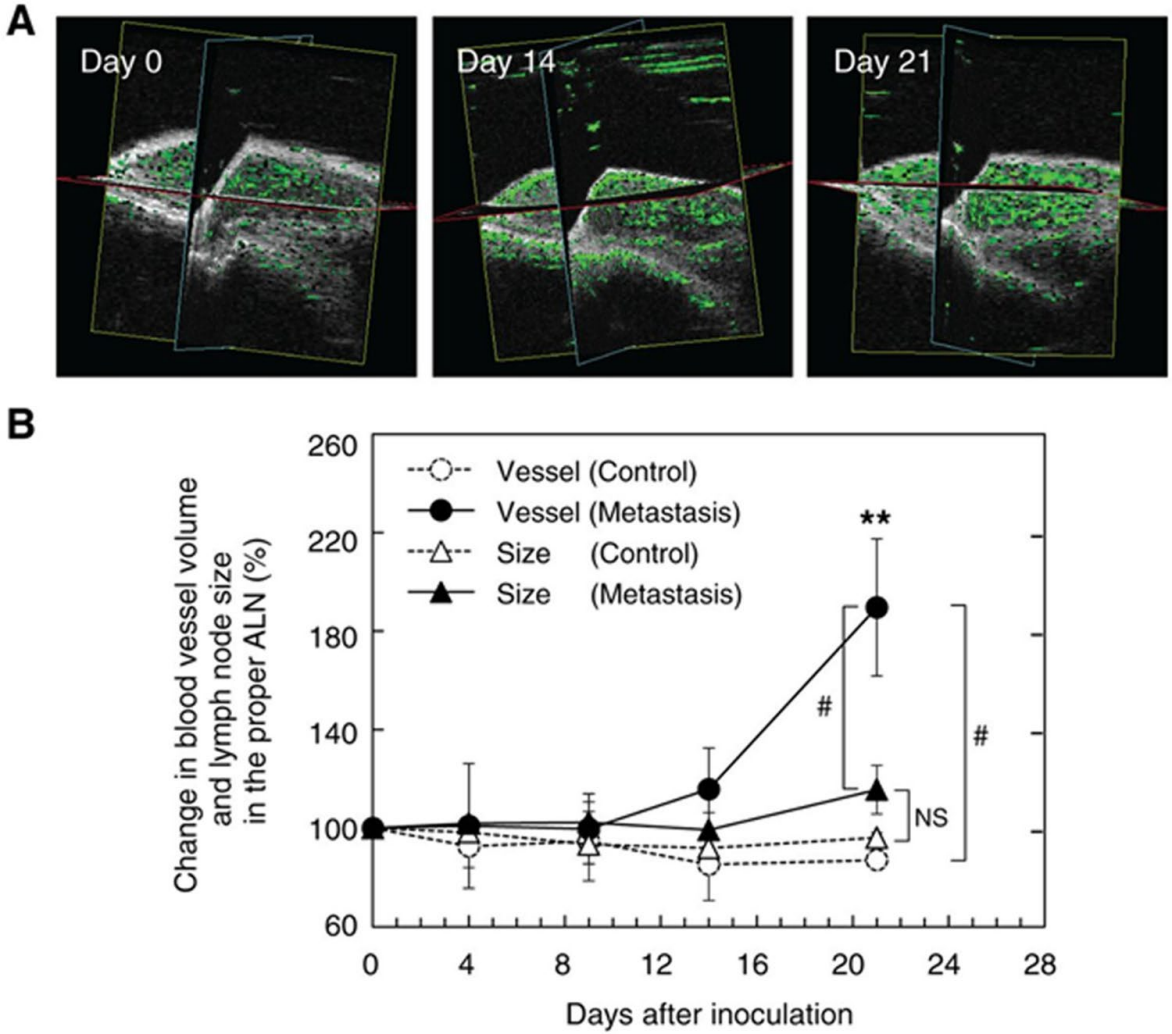
Changes in blood vessel volume and lymph node (LN) size of axillary LNs using contrast-enhanced ultrasound (CE-US). **A** The CE-US images to visualize the blood vessel structure (highlighted in green) of the axillary LN on days 0, 14, and 21. **B** changes in blood vessel volume and LN size. Control represents the axillary LNs without metastasis. The circles represent the blood vessel volumes within the axillary LNs of the control (○) and metastasis groups (●), respectively. The triangles represent the sizes of the axillary LNs of the control (△) and metastasis groups (▲), respectively. On day 21, the magnitude of the change in blood vessel volume was significantly larger than the magnitude of the change in lymph node size. Reprinted from Ref. 27 with permission

Animal studies of LN metastasis have been constrained by the limitations of the difficulty of establishing suitable animal models and the technology available for noninvasive monitoring of LN metastasis. Li et al. developed a mouse model of LN metastasis via afferent lymphatic vessels for the development of imaging modalities [31]. They used MRL/MpJ-/lpr/lpr (MRL/lpr) mice, which exhibit remarkable systemic lymphadenopathy and have axillary LNs and subiliac LNs as large as human LNs at 12 mm in diameter, respectively. Injection of KM-Luc/GFP malignant fibrous histiocyte-like cells into subiliac LNs allowed detection of metastases in axillary LNs within 3–9 days. Three-dimensional (3D) perfusion CE-US imaging showed that the microvascular volume and microvascular density of metastatic axillary LNs were significantly increased on day 14 after tumor cell injection into subiliac LNs [31]. Within 21 days after tumor cell injection into subiliac LNs, a significant increase in the microvascular volume and microvascular density of axillary LNs was observed, but no increase in the size of axillary LNs was observed (Fig. 3) [27]. The results of these studies indicate that changes in microvascular volume and microvascular density occur prior to changes in LN size in the early stages of LN metastasis. Recently, an LN metastasis model in large animals (swine) was successfully established, as the small body size of rodents such as mice is insufficient for measuring the lymphatic flow, applying new imaging techniques, determining the optimal dose of therapeutic agents, and determining the optimal treatment protocols [32]. Furthermore, Rowland et al. demonstrated that perfusion CE-US can be used to noninvasively visualize and quantify microvascular structures and blood flow dynamics using rabbit LNs and reported that perfusion CE-US has the potential to detect LN metastases in human clinical cases [33].

### 2–3. Differences between perfusion CE-US and lymphatic CE-US in the diagnosis of axillary LN metastases in breast cancer patients

**Fig. 4:**
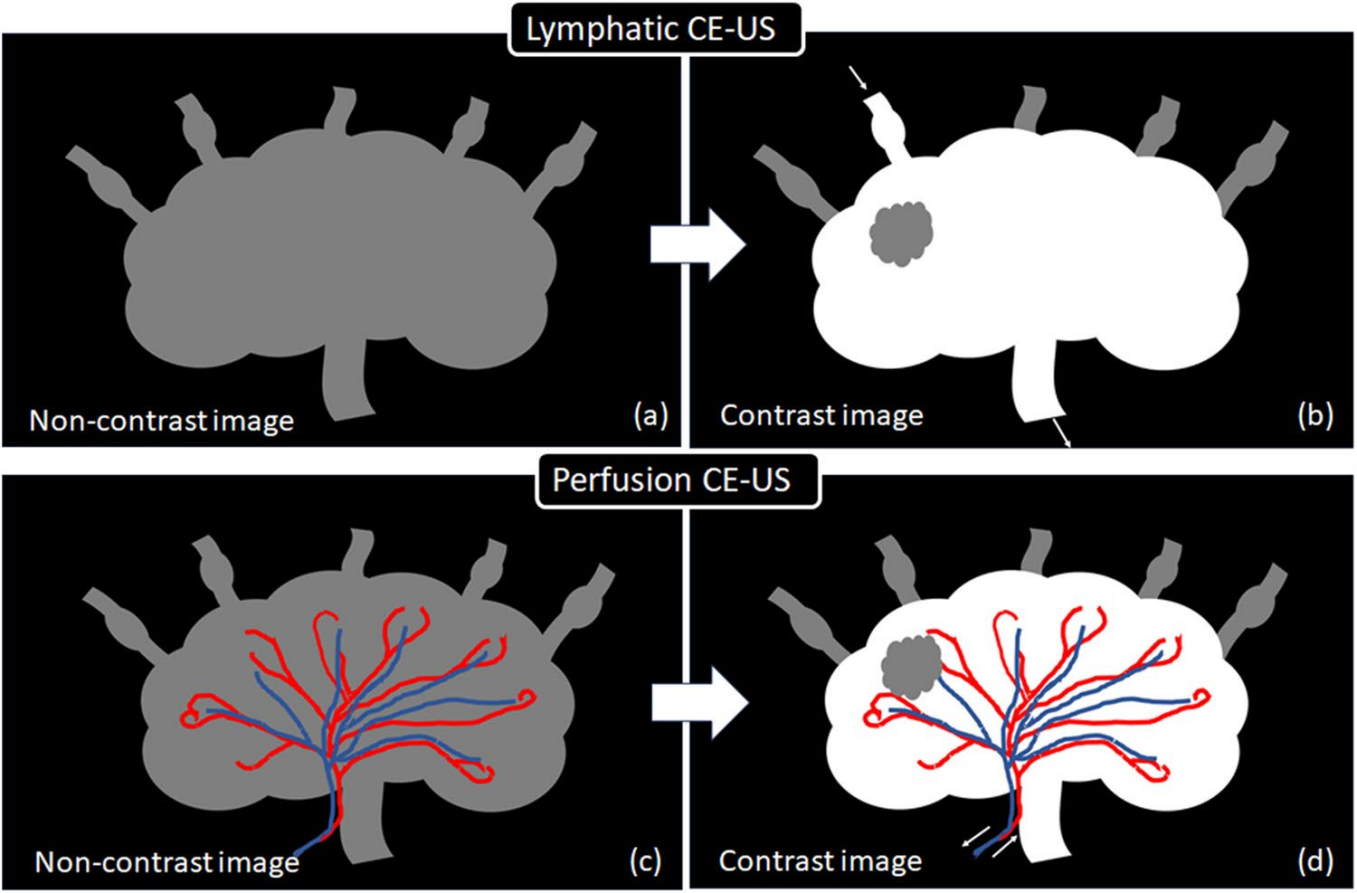
Mechanisms of detection of lymph node (LN) metastasis using lymphatic contrast-enhanced ultrasound (CE-US) and perfusion CE-US. In lymphatic CE-US, the contrast agent flows into the LN from the afferent lymphatic vessel on the capsule side, fills the LN, and is excreted from the efferent lymphatic vessel on the lymphatic hilum (**b**). Compared to the non-contrast image (**a**), the internal structures of normal LNs are contrast-enhanced, while the metastatic areas are not contrast-enhanced and become defects. In perfusion CE-US, the contrast agent enters the LN through the artery of the lymphatic hilum, perfuses the LN, and drains out through the vein of the lymphatic hilum. Compared to the non-contrast image (**c**), the internal structures of normal LNs are contrast-enhanced, while metastatic areas are not contrast-enhanced, resulting in a perfusion defect

LNs have two vasculatures, i.e., lymphatic and blood circulation, and tumor cell invasion into LNs affects the lymphatic and vascular circulation within the LNs. In animal experimental studies, tumor cells were transplanted from downstream LNs to upstream LNs via afferent lymphatic vessels, and contrast agents were intravenously administered via a vein in the tail of the mouse for imaging. In humans, both lymphatic CE-US and perfusion CE-US have been used in clinical studies. In lymphatic CE-US, the contrast agent reaches the sentinel LN via the lymphatic vessels, fills the LN, and is drained via the lymphatic vessels (Fig. 4). In perfusion CE-US, the contrast agent perfuses the LN through the artery at the lymphatic hilum and is drained through the vein at the lymphatic hilum (Fig. 4). Note that the LNs observed in perfusion CE-US are not always sentinel LNs. In 2002, Omoto et al. reported a new method for identifying sentinel LNs using CE-US in animal experiments [34]. Goldberg et al. then imitated Omoto’s method and introduced lymphatic CE-US as a noninvasive and reproducible method for detecting sentinel LNs [35]. Furthermore, Omoto et al. were the first to report the identification of sentinel LNs using Sonazoid in clinical breast cancer patients [36]. It allows real-time monitoring of the lymphatic vessels from the primary tumor to the sentinel LN after peritumoral injection of US contrast media [19]. Lymphatic CE-US has two purposes: detection of sentinel LNs and diagnosis of LN metastasis, with a detection performance reported to be 70% to 100% in a meta-analysis [37]. In 2009, the SLN identification rate with CE-US was reported to be 70% [36], while more recently it was reported to be quite high (90–98%) [19, 38, 39]. The diagnostic performance was reported to be more sensitive than that of perfusion CE-US (0.92 vs. 0.82, p < 0.05) in a meta-analysis [40]. Lymphatic CE-US has high diagnostic performance for LN metastases, but its detection performance for sentinel LNs is variable, and the question of whether to use perfusion CE-US or lymphatic CE-US is an important issue for future research [41].

### 2–4. Methods for evaluating axillary LN metastasis using perfusion CE-US

**Fig. 5:**
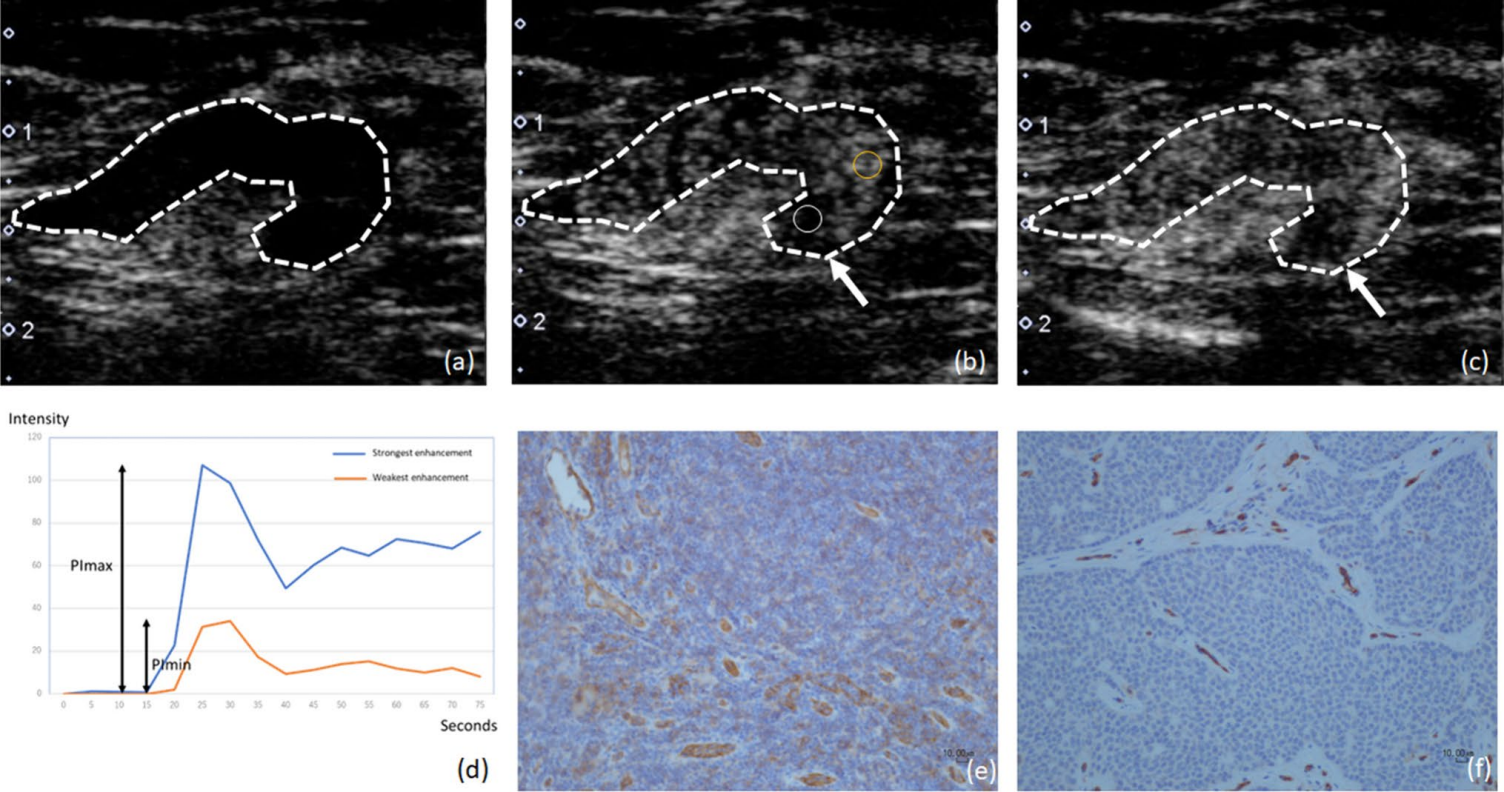
**A** 42-year-old woman preoperatively diagnosed with invasive breast carcinoma at biopsy. Perfusion contrast-enhanced ultrasound (CE-US) of a lymph node (LN) (dotted line) with the largest cross-sectional area showing heterogeneous enhancement (**b**) from the arterial phase (23 s after intravenous injection of contrast agent) to the venous phase (50 s after intravenous injection of contrast agent) (**c**) compared to the non-contrast image (**a**). The heterogeneous enhancement was interpreted as having perfusion defects (arrows) (**b**, **c**). The region of interest (ROI) was placed where the strongest and weakest enhancement were found (orange circle and white circle). Time–intensity curves (TICs) were generated from the ROIs with time on the x-axis and signal intensity on the y-axis. Peak intensity (PI) was measured from the TIC. The PI from the ROI with the strongest enhancement was recorded as PImax. The PI from the ROI with the weakest enhancement was recorded as PImin. Mouse anti-human CD31 monoclonal antibody (Dako Cytomation, Glostrup, Denmark) was used to evaluate the vascularity of the LN immunohistochemically (**e**, **f**). The area with metastatic deposits (**f**) was less vascularized than that with normal LN parenchyma (**e**) (× 200 field). The status of the LN was diagnosed as positive with a 12-mm metastatic nest at surgery

**Fig. 6:**
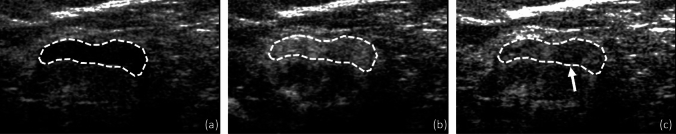
**A** 47-year-old woman preoperatively diagnosed with invasive breast carcinoma at biopsy. Perfusion contrast-enhanced ultrasound (CE-US) of a lymph node (LN) (dotted line) with the largest cross-sectional area showed almost homogeneous enhancement (**b**) in the arterial phase (23 s after intravenous injection of contrast agent) but heterogeneous enhancement in the venous phase (50 s after intravenous injection of contrast agent) (**c**) compared to the non-contrast image (**a**). The heterogeneous enhancement was interpreted as having perfusion defects (arrows) (**c**)

Visual and quantitative assessments have been introduced in the evaluation of perfusion CE-US of axillary LNs. Heterogeneity of contrast enhancement was used for visual evaluation [42, 43], and a combination of visual and quantitative assessment was used in the literature [44]. Zhuang et al. considered the timing of visual assessment and classified them into Type A: uniform enhancement in the arterial phase and uniform regression in the venous phase, and Type B: uniform enhancement in the arterial phase and heterogeneous regression in the venous phase [45]. In our institution, we observed a case of LN metastasis with heterogeneous contrast enhancement from the arterial phase and heterogeneous contrast enhancement in the venous phase, which appeared to indicate a perfusion defect (Fig. 5). On the other hand, there were cases of LN metastases that were homogeneously contrast-enhanced in the arterial phase and appeared heterogeneous with perfusion defects in the venous phase, in agreement with the report by Zhuang et al. (Fig. 6). Zhu et al. suggested that coarse or twisted penetrating vessels, rather than internal contrast enhancement, were useful in differentiating metastatic from non-metastatic LNs [46]. In contrast, several methods have been reported for quantitative analysis of perfusion CE-US in general [47]. Simple subtraction of US imaging before and after contrast enhancement is one method for detecting contrast agents [48, 49]. Temporal maximum-intensity-projection imaging [49, 50] and time-intensity curve (TIC) analysis after intravenous administration of contrast agents have also been proposed [51]. The region of interest (ROI) is placed where the enhancement was found. TIC is generated from the ROI with time on the x-axis and signal intensity on the y-axis (Fig. 5f). The subtraction method and temporal maximum-intensity-projection imaging can be severely distorted if the patient moves during the examination [50, 52]. On the other hand, TIC analysis is implemented in clinical US systems. Several studies have shown a correlation between parameters derived from TIC analysis of perfusion CE-US and histological microvessel density in the evaluation of breast cancer lesions [51, 53]. Pitre-Champagnat et al. reported that peak intensity and area under the curve of TIC analysis were significantly correlated with microvessel density in animal models [54]. The newly developed bubble detection method detects microbubble contrast agents in blood vessels by evaluating the changes in pixel intensity over a time axis and calculates the percentage of blood vessels filled with contrast agent in a given tissue volume (enhancement area ratio) [52]. The enhancement area ratio in the new bubble detection method correlates with histological microvessel density in human-invasive breast cancer [55]. These quantitative analyses of perfusion CE-US can potentially identify changes in microvascular density in axillary LNs.

### 2–5. Diagnostic performance of perfusion CE-US for axillary LN metastasis

Several studies have evaluated the diagnostic performance of perfusion CE-US for the evaluation of axillary LN metastases in patients with breast cancer, and it should be noted that these studies include different subject groups. The studies include apparently enlarged LNs [42–45] and clinical node-negative cases without apparently enlarged LNs on CT, MR, or conventional US [46, 56]. In studies with apparently enlarged LNs, the visual heterogeneity within LNs in perfusion CE-US showed sensitivity of 82–100%, specificity of 82–93%, and accuracy of 88–92% [42, 43, 45]. Du et al. reported that the maximum signal intensity, duration of contrast enhancement, and time to peak of the TIC analysis of perfusion CE-US were independent factors predicting axillary LN metastasis, with AUC of ROC curve analysis, sensitivity, and specificity being 0.936, 92%, and 87%, respectively [44]. In studies with clinical node-negative cases, one study using visual coarse or twisting penetrating vessels as the criterion had an AUC of ROC curve analysis of 0.72 [46]. In our study, maximum peak intensity (PImax) and minimum PI (PImin) were obtained by placing the ROI at the strongest and weakest signal increases in the TIC analysis, and their ratio was calculated as PI ratio. The sensitivity and specificity of PI ratio were 59% and 87%, respectively (Fig. 5f) [56]. Perfusion CE-US showed heterogeneous enhancement with an obvious perfusion defect. Mouse anti-human CD31 monoclonal antibody (Dako Cytomation, Glostrup, Denmark) was used to evaluate the microvessel density of LNs immunohistochemically. The area with metastatic deposits was less vascularized than that with normal LN parenchyma (Fig. 5e and f). In clinical node-negative cases, the diagnostic performance of perfusion CE-US for LN metastases is lower than that for apparently enlarged LNs due to the difficulty in detecting small early metastases. Perfusion CE-US is required to detect small early LN metastases, and it is expected that the development of 3D analysis of CE-US, its evaluation method (visual or quantitative), and evaluation parameters for quantitative analysis will be standardized in the future to improve the diagnostic performance.

### 2–6. Limitation of perfusion CE-US compared to lymphatic CE-US

The disadvantage of US including perfusion CE-US and lymphatic CE-US is that it mainly uses two-dimensional (2D) images, making it difficult to obtain an overview of the entire axillary region in which the suspected LN metastases are located and operator-dependent [10]. Perfusion CE-US can only be performed by selecting a LN with the largest cross-sectional area, unless combined with lymphatic CE-US or dye-based sentinel LN identification. Consequently, even if the selected LN is suspected to have metastasis based on perfusion CE-US, it does not always mean that the selected LN corresponds to a sentinel LN, and therefore, it is not known whether the sentinel LN has metastasis or not. In addition, since perfusion CE-US targets a single LN, only one node can be evaluated at a time as long as a 2D transducer is used. If there are multiple suspicious LNs in the axilla, the contrast agent would need to be administered in divided doses.

### 2–7. Challenges of CE-US in the coronavirus disease 2019 (COVID-19) pandemic era

Currently, the coronavirus disease 2019 (COVID-19) vaccine is administered worldwide, and after vaccination, regional lymphadenopathy in the axillary and supraclavicular regions has been reported with a prevalence rate of 1.1% and is referred to as COVID-19 vaccine-associated lymphadenopathy [57]. Such reactive enlarged LNs, including COVID-19 vaccine-associated lymphadenopathy, are difficult to distinguish from metastatic LNs because of the increased short diameter and thickness of the thickest part of the cortex. Future studies are needed to determine whether perfusion CE-US and lymphatic CE-US can differentiate COVID-19 vaccine-related lymphadenopathy from metastatic LNs.

### 2–8. Diagnosis of LN metastasis with perfusion CE-US in areas other than breast cancer

**Fig. 7:**
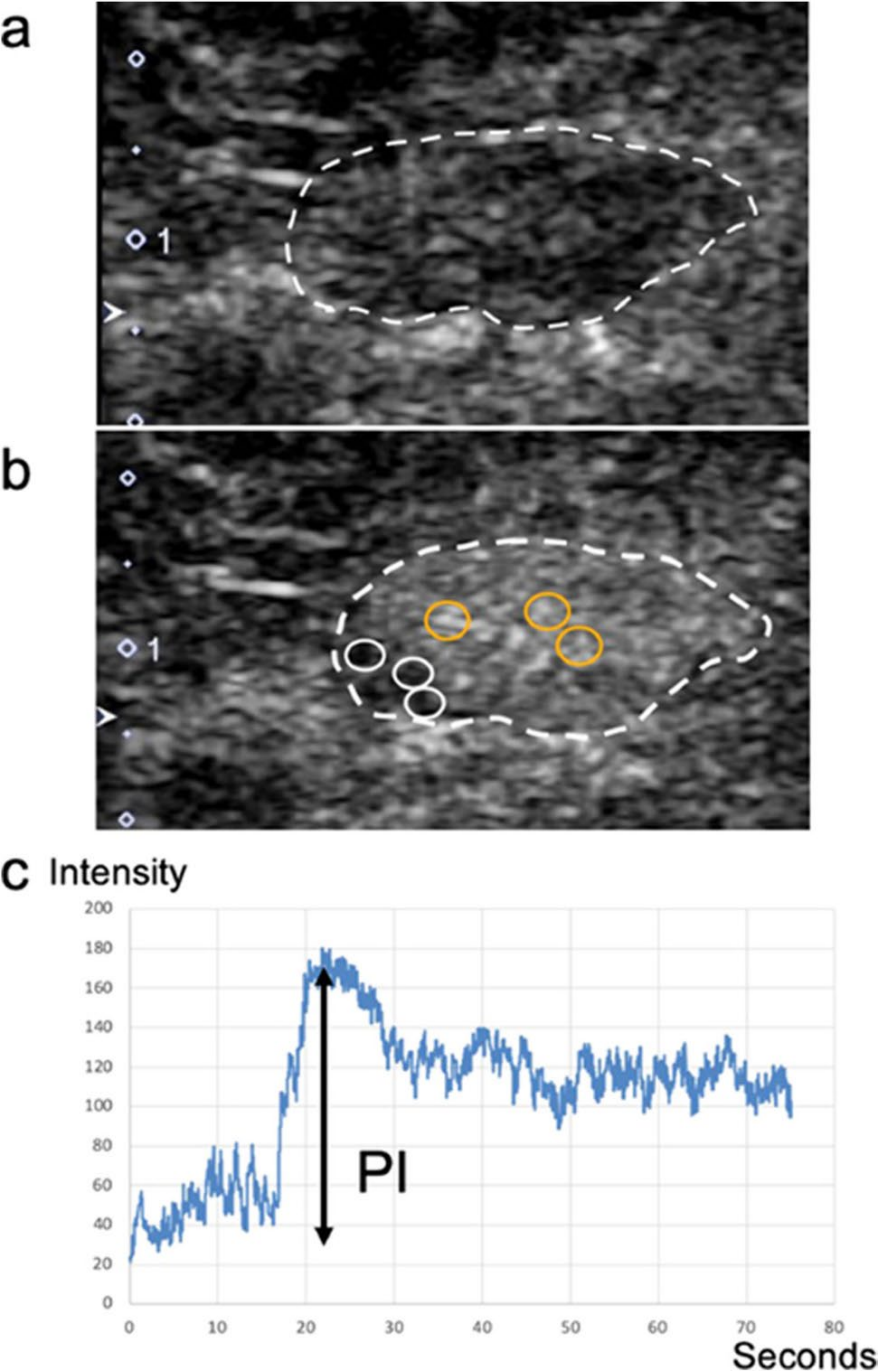
Methods for placing regions of interest (ROIs) in sentinel lymph nodes (LNs) and acquiring time-intensity curves (TICs) for intraoperative perfusion contrast-enhanced ultrasound (CE-US) in uterine cancer. **a** Intraoperative pre-contrast US revealing manual segmentation covering the entire LN volume (dotted line). **b** Intraoperative perfusion CE-US at the arterial phase. Inside the LN (dotted line), three ROIs (orange) were selectively placed where the strongest enhancement was observed. The other three ROIs (white) were selectively placed where the weakest enhancement was observed. **c** TICs were generated from the ROIs with time on the x-axis and signal intensity on the y-axis. Peak intensity (PI) was measured from the TIC. The maximum PI value from the three ROIs with the strongest enhancement was recorded as PImax. The minimum PI value from the three ROIs with the weakest enhancement was recorded as PImin. Reprinted from Ref. 65 with permission

**Fig. 8:**
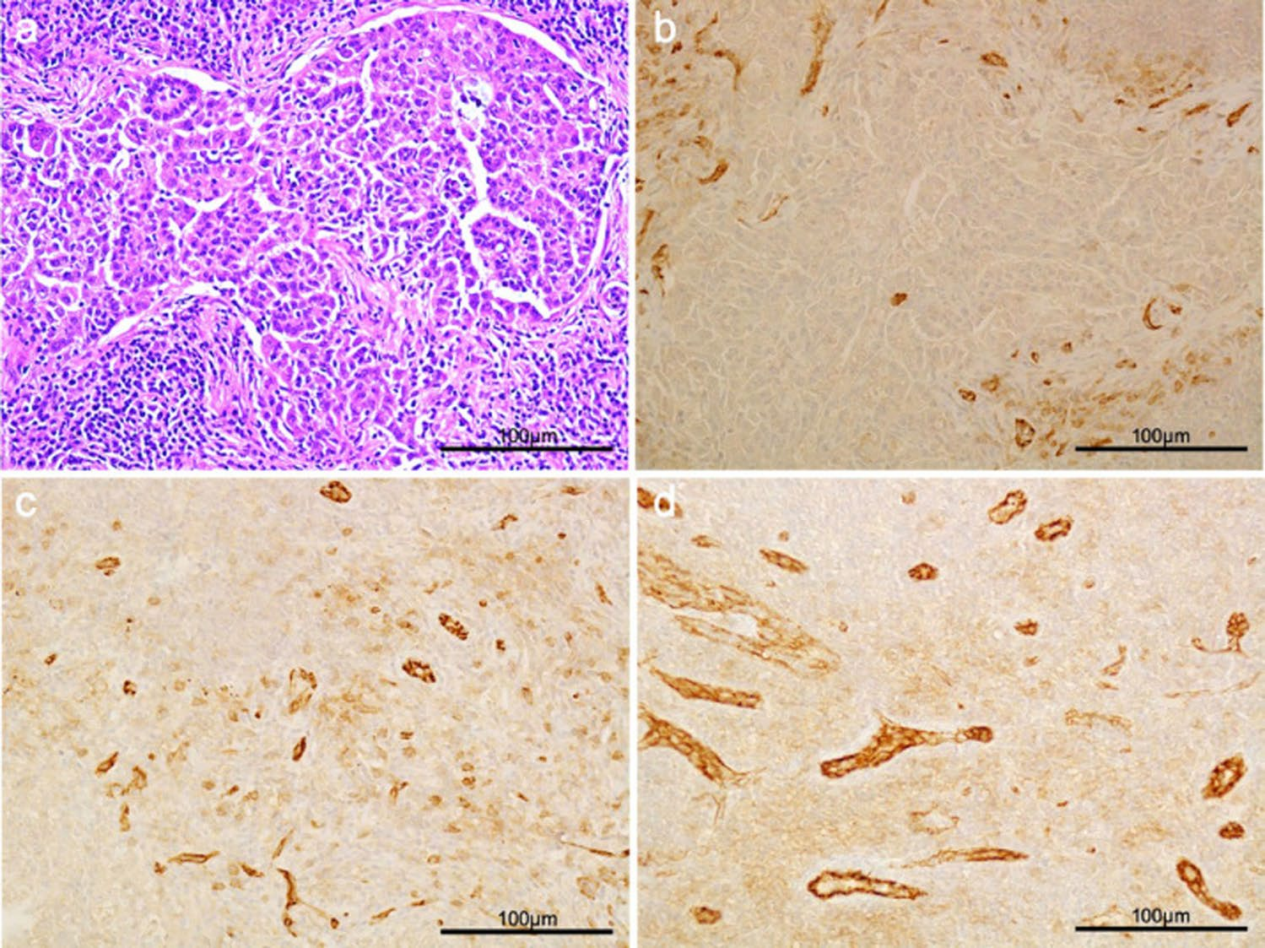
Analyses of microvessel density in a resected left obturator sentinel LN of a 58-year-old woman with uterine endometrial cancer. The sentinel LN exhibited a 2.3-mm metastasis on hematoxylin and eosin staining (**a**). The area percentage of CD31-immunostained vascular structures per field was calculated in the metastatic lesion (**b**), around the metastatic lesion (**c**), and in normal parenchyma (**d**) using the “hot-spot” method under × 200 magnification. The microvessel density of metastatic lesions was lower than that of other fields. Reprinted from Ref. 65 with permission

Perfusion CE-US has been applied to the diagnosis of LN metastasis in various areas in clinical studies. There have been several reports of diagnosing cervical LN metastases using perfusion CE-US in patients with papillary thyroid carcinoma [58, 59] and squamous cell carcinoma of the oral cavity [60]. Perfusion CE-US has also been used in the diagnosis of LN metastasis in cutaneous melanoma [61–63]. All these studies involved superficial primary lesions and LNs and shared the common feature that imaging with perfusion CE-US was available. There are also reports of intraoperative perfusion CE-US of deep LNs (in the pelvis) in uterine cancer, where a perfusion defect was associated with the presence of metastases [64]. Toki et al. detected pelvic sentinel LNs through cervical injections of dye and/or radioisotopes and then placed US probes directly over the LNs to perform CE-US. The PI ratio in the TIC analysis of the strongest and weakest enhancement areas was significantly different between the metastatic and non-metastatic LN groups (Fig. 7). They also confirmed that the microvascular density of metastatic lesions was significantly lower than that of other normal sites within LNs (Fig. 8). The strength of the study by Toki et al. is that even with perfusion CE-US, selective perfusion CE-US of sentinel LNs could be performed by combining it with intraoperative sentinel LN identification. Intraoperative perfusion CE-US may also be useful in the diagnosis of deep LN metastases [65]. Originally, Rubaltelli et al. reported in 2004 that perfusion defects with perfusion CE-US in LNs of squamous cell carcinoma of the head and neck, cutaneous melanoma, breast cancer, and rectal cancer were useful in the diagnosis of LN metastases [63]. They further reported that the difference between the maximum and minimum signal intensity of the automated quantification software was useful in distinguishing benign from malignant LNs in cervical squamous cell carcinoma, cutaneous melanoma, and breast cancer [62]. These results of Rubaltelli et al. have been applied to the diagnosis of axillary LN metastasis in breast cancer, and the method is being brushed up and further applied to LN metastasis in other superficial head and neck cancers and deep-seated cancers.

## Conclusion

In summary, we reviewed the significance of axillary LN metastasis in breast cancer, discussed the limitations of conventional US, and highlighted the potential of perfusion CE-US as a diagnostic tool. We showed the characteristics of contrast agents in CE-US, animal models for LN metastasis, differences between perfusion CE-US and lymphatic CE-US, and the diagnostic performance of perfusion CE-US for axillary LN metastasis in breast cancer, as well as its application in other cancer types.
